# Correction

**Published:** 1883-02

**Authors:** 


CORRECTION.—Owing to an unfortunate error in the January number, the following selected articles appeared without proper credit to our exchanges in which they originally appeared:

"Locomotor Ataxia, Case," by A. M. Carpenter, from the *Indiana Med. Reporter.*

"The Bradshawe Lecture," by Sir James Paget.

"Abdominal Section," by J. Ewing Mears, Philadelphia.

"Notes on Therapeutics," by R. L. MacDonnell, Montreal.

"Self-Limited Duration of Pulmonary Phthisis," by Austin Flint, N. Y.

"Puerperal Fever," by John Lowe, Litchfield, Eng.

"Dry Cupping and Rest in Locomotor Ataxia," H. M. Lyman.

